# Work Characteristics Associated with Physical Functioning in Women

**DOI:** 10.3390/ijerph14040424

**Published:** 2017-04-15

**Authors:** Aimee J. Palumbo, Anneclaire J. De Roos, Carolyn Cannuscio, Lucy Robinson, Jana Mossey, Julie Weitlauf, Lorena Garcia, Robert Wallace, Yvonne Michael

**Affiliations:** 1 Department of Epidemiology and Biostatistics, Dornsife School of Public Health, Drexel University, Philadelphia, PA 19104, USA; lfr32@drexel.edu (L.R.); jm55@drexel.edu (J.M.); ylm23@drexel.edu (Y.M.); 2 Department of Environmental and Occupational Health, Dornsife School of Public Health, Drexel University, Philadelphia, PA 19104, USA; ajd335@drexel.edu; 3 Section on Public Health, Perelman School of Medicine, University of Pennsylvania, Philadelphia, PA 19104, USA; cannusci@wharton.upenn.edu; 4 Veterans Affairs, Palo Alto Health Care System, Palo Alto, CA 94304, USA; wjulie1@stanford.edu; 5 Department of Psychiatry and Behavioral Sciences, Stanford University School of Medicine, Stanford, CA 94305, USA; 6 Department of Public Health Sciences, UC Davis School of Medicine, Sacramento, CA 95616, USA; lgarcia@ucdavis.edu; 7 Department of Epidemiology, University of Iowa, Iowa City, IA 52242, USA; Robert-wallace@uiowa.edu

**Keywords:** workplace, social environment, women’s health, physical function

## Abstract

Women make up almost half of the labor force with older women becoming a growing segment of the population. Work characteristics influence physical functioning and women are at particular risk for physical limitations. However, little research has explored the effects of work characteristics on women’s physical functioning. U.S. women between the ages of 50 and 79 were enrolled in the Women’s Health Initiative Observational Study between 1993 and 1998. Women provided job titles and years worked at their three longest-held jobs (*n* = 79,147). Jobs were linked to characteristics in the Occupational Information Network. Three categories of job characteristics related to substantive complexity, physical demand, and social collaboration emerged. The association between job characteristics and physical limitations in later life, measured using a SF-36 Physical Functioning score <25th percentile, was examined using modified Poisson regression. After controlling for confounding variables, high physical demand was positively associated with physical limitations (RR = 1.09 CI: 1.06–1.12) and substantively complex work was negatively associated (RR = 0.94, CI: 0.91–0.96). Jobs requiring complex problem solving, active learning, and critical thinking were associated with better physical functioning. Employers should explore opportunities to reduce strain from physically demanding jobs and incorporate substantively complex tasks into women’s work to improve long-term health.

## 1. Introduction

The U.S. Census Bureau estimates that the population aged 65 and over will double by 2050 and will account for over 20% of all U.S. residents [1]. The oldest-old age group, those aged 85 and older, is expected to grow to 18 million and account for 4.5% of the population [1]. As the population ages, an increasing proportion of adults will experience disease and disability and require medical care or supportive services. In 2012, 28% of adults 65–74 and 45% of adults 75 and over had difficulty performing physical activities and women were more likely to have difficulties than men, even after adjusting for age [2]. Limitations in physical functioning are restrictions in basic physical activities of daily living and are an intermediate step on the theoretical pathway to disability [3,4]. Chronic conditions, in addition to injuries and disuse, cause the majority of disability in older adults; however, the pathway is complex and often influenced by a multitude of disease and non-disease factors [5]. 

Work is a potential contributor on the pathway to disability. One mechanism through which work influences physical functioning is the psychosocial environment of work [6]. Psychosocial work exposures have been conceptualized in a number of ways including job strain, effort-reward imbalance, and social support [7,8,9,10]. These conceptual models posit that constant exposure to work environments, which are shaped by the level of work load demands, decision latitude, and social interactions, are embodied by individuals and influence their health [7,8,9]. The relationship between psychosocial exposures at work and health has been well documented [8,11,12,13]. Recent meta-analyses consistently found job strain to be associated with increased risk of poor health outcomes such as coronary heart disease and hypertension [14,15], poor mental health outcomes such as depression [16], and the development of musculoskeletal problems [6]. All of these health outcomes are important components of, and contributors to, physical functioning. The WHO recognizes that work atmosphere is a critical contributor to overall health, with poor psychosocial work characteristics increasing the risk of poor mental and physical health outcomes [17,18,19]. At the same time, social support at work has been shown to be independently associated with improved work experience and health outcomes such as better physical functioning [20,21]. 

Women now make up almost half of the labor force and the percentage of women with college degrees has more than tripled since 1970 [22,23]. In the second half of the 20th century, women shifted away from traditionally female occupations such as clerical work, teaching, and nursing, to occupations such as managers, administrators, accountants, lawyers, and physicians [24]. Despite this shift in labor force participation, few research studies have explored the long-term effect of the psychosocial work environment on women’s long-term health. Two studies with 4–5 years of follow-up found that women with self-reported high job demands and low social support at work were at increased risk for poor physical functioning [21,25]. A systematic review of studies on psychosocial work stress and musculoskeletal problems in industrialized countries published through 2009 found that of the 45 high-quality studies included in the analysis, only three had follow-up periods of 10 years or more and these studies were all conducted in Scandinavian countries [6]. While women were included in these studies, only one of the three studies with substantial follow-up conducted separate analyses by gender, finding that dissatisfaction with work control and social relationships at work were associated with a 10-year increase in musculoskeletal morbidity in both white-collar and blue-collar women workers [26]. A better understanding of work’s influence on physical functioning in women is necessary, given women’s substantial participation in the work force [27,28]. 

Jobs with high demands and low rewards may be associated with decreased physical functioning [6]. Previous studies, such as the Whitehall II study, have found that workers in higher grade jobs had higher proportions of beneficial work characteristics such as high control, varied work, and high satisfaction than workers in lower grade jobs [13], and higher grade jobs were also associated with better physical functioning [25]. These findings are consistent with Karasek’s job classifications, where higher status jobs such as architects, scientists, and programmers tended to have more favorable characteristics (higher control, lower demands) and lower status jobs such as assemblers, garment stitchers, and telephone operators had less favorable characteristics [29].

The relationship between work and health may be explored through the context of life course epidemiology, which acknowledges that extrinsic factors or stressors are embodied throughout a person’s life; the effects of these factors accumulate over time and critical periods are specific time periods during the life course in which factors have a greater influence on health [30]. The critical period model, which emphasizes the timing of exposures, has been expanded to demonstrate that socioeconomic position, a concept inherently linked to occupation, throughout the life course impacts later life health [31,32,33]. Using a life course framework, the impact of work characteristics on health may depend on exposure during critical periods, such as during times of giving birth and taking care of small children [30,34]. This study aims to explore the effects of work characteristics, overall and at critical periods in women’s lives, on later life physical functioning. This study expands on previous studies through our ability to analyze work characteristics during critical time periods. We expect that women in jobs with adverse work characteristics will be more likely to experience functional limitations than women in jobs with beneficial characteristics; we expect these effects to be more pronounced during the time period when women have young children.

## 2. Materials and Methods

### 2.1. Study Population

Women between the ages of 50 and 79 were enrolled in the Women’s Health Initiative Observational Study (WHI-OS) between 1993 and 1998 (*n* = 93,676) [35]. The study details have been described elsewhere [35,36]. The eligibility criteria for WHI-OS included age over 50 years, postmenopausal status, willingness to provide informed consent, and at least a 3-year life expectancy [37]. At baseline, women provided abbreviated work history information, demographic information, information about lifestyle and behavioral exposures such as tobacco and alcohol use that are important risk factors for later life health, information on medical conditions and treatments, and self-assessment of their current physical functioning. All women in the WHI-OS who reported working at least one job outside of the home were eligible to be included in this study (*n* = 91,627), including women who primarily considered themselves homemakers but still reported a job outside the home. Women were excluded if they had missing values for job details (job title and duration, age when job started) or for physical functioning. Women were also excluded if they reported working only in military jobs, as job exposure information for military occupations was unavailable in our exposure matrix. Ethical approval for this study was provided by Drexel’s IRB. 

### 2.2. Exposure Assessment

Women provided job title, industry, age at which job began, and total duration of employment for up to three paid jobs held the longest since age 18. Unstructured text fields of job title and industry were matched using a computer algorithm to 2010 Standard Occupational Classification (SOC) codes, a system used by Federal agencies to classify workers into occupational categories, at the highest level of detail available [38]. Precision of the match (percent agreement) was evaluated using a comparison to a subset of manually coded occupations and was found to be adequate, ranging in agreement from 0.72 (or “moderate”) for ”broad occupation” groups (the first 4–5 digits in SOC codes) to 0.85 (“very good”) for “minor groups” (the first 3 digits in SOC codes) [39]. Standard codes for job titles were linked to characteristics in the Occupational Information Network (O*NET), a database of occupational descriptions widely used to identify characteristics and demands of jobs [40]. These job characteristics served as the primary exposure variables for our analysis and data for 858 detailed job titles (6-digit SOC codes) were available from O*NET version 18.1 for worker characteristics related to worker abilities, worker interests, and work styles; for worker requirements related to knowledge and skills; and for occupational requirements related to work context and activities [40]. At higher levels of SOC codes (e.g., 3-digit), values were averaged across all detailed jobs that fell within that level. O*NET rating values had different scales, depending on the category; values for all characteristics were transformed to a range between 0 and 1. For categories related to worker abilities, worker knowledge and skills, and work activities, variables were available which described both the level, or the “’extent’ to which a value applies to an occupation”, based on ratings by subject matter experts, and the importance, indicating “the rating of importance for an item”, for each job [40]. For these variables, since level and importance are highly correlated, the values were averaged together to obtain one score for each characteristic [41]. Other categories had single values available for analysis. 

### 2.3. Outcome Assessment

Self-reported physical functioning at baseline was assessed using the Physical Functioning (PF) scale on the Rand 36-Item Health Survey (SF-36) [42,43]. The scale is based on responses to 10 questions related to physical activities from vigorous activities such as running to moderate and lighter activities such as climbing stairs and walking, and whether respondents are limited a lot, a little, or not at all. The scale was designed to be applicable to both general populations and those with health conditions and has been shown to have a high internal-consistency reliability across diverse groups (Cronbach’s alpha = 0.90) [43,44]. Responses to questions were scored as 1 to 3, respectively, and were then summed and transformed to a scale of 0 to 100 where a low score represented a lot of limitations in performing all physical activities, including bathing and dressing, and a high score represented no limitations in all types of physical activities [44]. Health-related quality of life varies according to age, gender, and measures, and the physical functioning variable is inherently non-normal, so the presence of physical limitations was determined based on cutoffs at the 25th percentile of the study population [45]. 

Factor analysis using promax rotation on all 241 available O*NET variables was conducted to identify categories of co-occurring job characteristics, replicating the methods used by both Hadden and Meyer [41,46]. Variables with factor loading values greater than 0.8 were retained and used to create a weighted average score of each factor, or job characteristic, for each job. Factor scores were averaged for each woman, across jobs, and weighted by the years at each job overall and during 4 distinct, critical periods of women’s adult lives: (1) before having children; (2) with young children (<age 5), (3) with older children (ages 5–18), and (4) after children became adults (>age 18). Work exposure periods for women without children were classified as period 1 for all jobs. A woman’s ‘exposure’ to a job characteristic was based on scores greater than or equal to the median value for each factor. We also evaluated the sensitivity of the results to the 25th and 75th percentile cutoff in factor scores.

### 2.4. Statistical Analysis

Modified Poisson regression was used to examine the association between the binary indicators of job characteristics in relation to physical limitations (PF score <25th percentile) [47]. Examining the association of job characteristics with a PF score <10th percentile and <50th percentile tested the sensitivity of results to the cutoff in physical functioning score. To understand whether job characteristics had a stronger influence on physical functioning during potentially critical periods of time, we derived a binary indicator variable of exposure to each job characteristic for each time period using the overall median value as the cutoff. 

A variety of other sensitivity analyses were conducted to evaluate potential bias due to the characteristics of our study population. To evaluate the presence of the healthy worker effect, or the possibility that women who left the work force earlier due to poor health may bias our results, we conducted an additional analysis limiting the population to women who were working at the WHI-OS baseline. Additionally, inherent differences may exist for women who self-identify as homemakers, as these women may have a preference for domestic work and may be more acutely impacted by workplace stressors. We conducted a separate analysis to assess whether the associations observed in the total population differed in this group of women. Finally, women veterans may have unique occupational exposures and a separate analysis was also conducted in this population. Although women who reported only military service were excluded from analysis, most women who served in the military also worked in civilian jobs. These women, half of whom were aged 70 or older at baseline, may have different reactions to the workplace environment, given their extreme minority status while serving in the armed forces, post-military service stigmatization, and historical restrictions on marriage and pregnancy [48].

Potential confounding variables were assessed for independent associations with both substantial physical limitations and with exposure to job characteristics. Inclusion of confounding variables in the final model was determined based on change in effect estimate of the exposure as well as statistical significance and theoretical considerations. We anticipated that education would have a uniquely important relation to work characteristics; in previous studies it was positively correlated with decision latitude and negatively correlated with physical demand [29,49]. This may be because education is so strongly tied to job characteristics and the type of jobs held throughout life may be largely dependent on the level of education. Furthermore, additional education is associated with later age at first birth [50], allowing women time to invest in and potentially initiate a career prior to starting a family. Therefore, education was assessed separately from the other confounders. Variables that could theoretically exist on the pathway between job characteristics and physical functioning were considered potential mediators and were analyzed separately in our models. All analyses were conducted using SAS 9.3.

## 3. Results

### 3.1. Study Population

**Figure 1:**
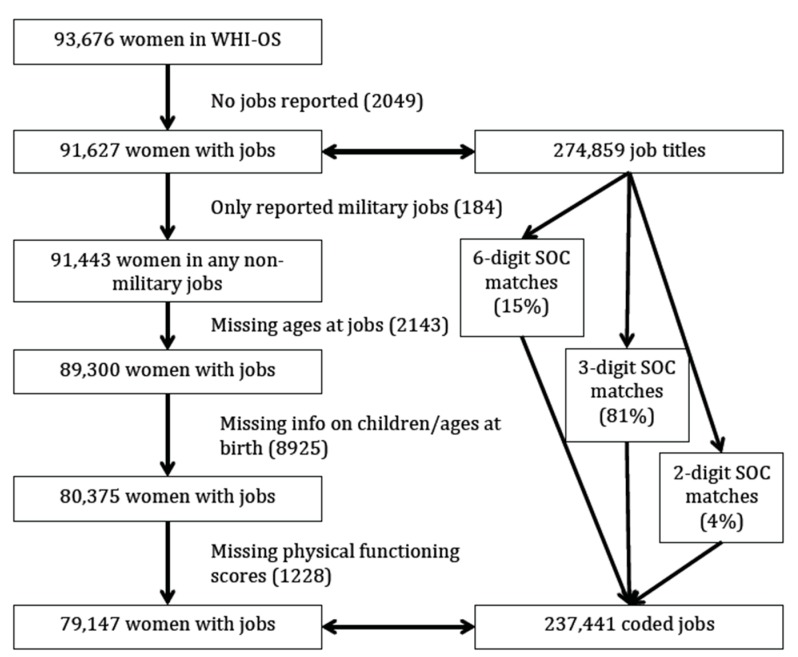
Diagram of the study population.

There were 274,859 job titles reported to WHI-OS by 91,627 women. Using the computer algorithm, 15% of the job titles were matched to 747 6-digit SOC codes, 81% were matched to 93 3-digit SOC codes and the remaining jobs were matched to 2-digit SOC codes. Only 61 job titles among 58 women were not matched to any SOC code. Military jobs have no associated O*NET values; 184 women whose majority of work was in military jobs were excluded. However, 1978 women who reported at least 180 days of military service also reported other work and were included in the analysis (2.7%). An additional 8925 women were missing information about the number of children or ages at first and last birth; 2143 women did not provide age at jobs; 1228 women had missing physical functioning scores. The final study population included 79,147 women with jobs representing all 22 major (2-digit) categories available in the SOC system (see Figure 1). 

### 3.2. Factor Analysis

Factor analysis of the job characteristic variables found 22 factors with Eigenvalues greater than 1, indicating that up to 22 unique factors could be extracted; scree plots showed a substantial drop in Eigenvalues after three factors, so three factors were retained as job characteristics. The first factor explained 38.5% of the variance and represented the work characteristic “substantive complexity,” where the highest loading variables were complex problem solving, active learning, decision making, deductive reasoning, analyzing information, and critical thinking (Table 1). The second factor explained 16% of the variance and represented the characteristic “physical demand,” where the highest loading variables were rate control, response orientation, and reaction time (Table 1). The third factor explained 8% of the variance and represented “social collaboration,” where the highest loading variables were self-control, concern for others, and social orientation (Table 1). The overall job scores for substantive complexity ranged between 0.032 and 0.65, with a median value of 0.48; the scores for physical demand were right skewed and ranged between 0.014 and 0.59, with a median value of 0.18; scores for social collaboration ranged between 0 and 0.96, with a median value of 0.75.

### 3.3. Outcome Analysis

Values for physical functioning (PF) were highly skewed to the left and ranged from 0 to 100 with a median value of 90, a 25th percentile value of 75, and a 10th percentile value of 50; a score below 75 was the main cutoff used to indicate the presence of physical limitations. Among the women in the bottom 25 percent of physical functioning, most had at least some limitations in performing vigorous activities such as running or lifting heavy objects (95%); climbing several flights of stairs (89%); bending, kneeling, or stooping (86%); and walking a mile (81%). 

The average age of participants at the WHI-OS baseline was 63; most women (61%) were married at baseline and the majority of study participants (86%) were white. Almost half of the women (43%) were still working at baseline and 26% considered themselves homemakers (Table 2). The most common jobs held overall by women in this sample were office and administrative support jobs (*n* = 15,678, 20%) and education, training, and library jobs (*n* = 14,425, 18%). The majority of women in education jobs (88%) were in positions that were substantively complex, whereas the majority of women in administrative support jobs (88%) were not. Women who held jobs with higher levels of substantive complexity (Factor 1 score ≥0.48) were more likely to have a college degree or higher (*p* < 0.01), more likely to be of normal weight (*p* < 0.01), and have higher family income (*p* < 0.01) compared to women who held jobs with low levels of substantive complexity (Table 2). Women with substantively complex jobs were slightly more likely to have worked before children (*p* < 0.01) and less likely to work in male-dominated and/or manual labor jobs that also include the most dangerous and accident-prone jobs (e.g., protective services, farming, fishing and forestry, construction and extraction) or in female-dominated jobs considered lower in socioeconomic status (SES) (e.g., food preparation and service) (*p* < 0.01) (Table 3). 

Women who held jobs with high physical demands (Factor 2 score ≥ 0.18) were more likely to have less than a high school degree (*p* < 0.01) and to have lower income (*p* < 0.01) than women in jobs with lower physical demand (Table 2). These women were also more likely to have held at least three jobs in their adult life (*p* < 0.01) and were more likely to work in male-dominated, manual labor jobs, but were also more likely to work in female-dominated jobs of lower SES (*p* < 0.01) (Table 3). Women who held jobs with high social collaboration (Factor 3 score ≥ 0.62) were more likely to list only one job (*p* < 0.01), were slightly more likely to work before having children (*p* < 0.01), and were more likely to work in high SES, female-dominated jobs (e.g., healthcare practitioners and support) (*p* < 0.01) than women who held jobs with lower social collaboration (Table 3). 

The analysis of interaction between critical periods and job characteristics showed that period-specific effects of job characteristics were important predictors of physical functioning in the crude model, but failed to substantially improve the model fit and were not statistically significant when confounders were added to the model. Thus, the results of work characteristics are reported without reference to critical period. Table 4 shows the results of regression analysis examining the association between job characteristics and physical limitations. After adjusting for confounders of age, birth region, race/ethnicity, and marital status, high substantive complexity and social support at work were inversely associated with physical limitations, compared to lower substantive complexity (RR = 0.81, CI: 0.79–0.84) and social support (RR = 0.94, CI: 0.92 = 0.97) at work. High compared to low physical demand was positively associated with physical limitations (RR = 1.12, CI: 1.10–1.15). However, after controlling for education, these results were attenuated, though in the same direction; substantive complexity was negatively associated with physical limitations (RR = 0.94, CI: 0.91–0.96), high physical demand was positively associated with limitations (RR = 1.09 CI: 1.06–1.12), and social support did not have a statistically significant association with physical limitations (Table 4, model 3). For physical demand, a small though statistically significant positive association with physical limitations remained even after adjusting for potential mediators (RR = 1.04 CI: 1.01–1.06). 

### 3.4. Sensitivity Analysis

Sensitivity analysis was conducted to understand the impact of the median cutoff in work characteristic scores in defining exposure. Associations remained substantively unchanged using the 25th or 75th percentiles to assess exposure for all three work factors. Additional analysis was also conducted using an alternative cutoff to define the outcome of physical limitations. Using the 50th percentile as the cutoff point (PF < 90), the results were similar to the results for the 25th percentile, but of smaller magnitude; using the 10th percentile (PF < 50), the results were in the same direction, but further from the null. After limiting the analysis to women actively working at baseline, the associations with work characteristics remained essentially unchanged, though moved slightly further from the null; however, in this population, working during the period with young children was significantly positively associated with physical limitations (RR = 1.07, CI: 1.02–1.13) and working in the period after children was no longer statistically significant (Table 4 and Table 5, Sensitivity Analysis). Estimates were similar when the analysis was limited to women who did not self-identify as homemakers. For women who identified as homemakers, the estimates of association between work characteristics and physical functioning were attenuated (Table 4, Homemakers); estimates of association with potentially critical time periods remained the same except for working during the time period with older children in the house, which became associated with a significant 9% increased probability of physical functioning limitations (Table 5, Homemakers). Limiting the analysis to veterans, confidence intervals became wide and were no longer statistically significant; point estimates were mostly similar to the main analysis, except for substantively complex jobs and working after children are grown, which had null estimates of association with physical limitations among veterans.

While we did not observe a statistically significant interaction between critical period and work characteristics, we did observe overall effects of working during specific time periods on physical functioning. Working in time period 4, after children become adults, was negatively associated with a 13% decreased risk of physical limitations (RR = 0.87, CI: 0.84–0.90), even after controlling for work in other time periods and other confounders, including age and education (Table 5, Model 3). We also found that working in time period 1, before having children, was negatively associated with an 8% decreased risk of physical limitations (RR = 0.92, CI: 0.89–0.94). These associations remained even after excluding the 14% (*n* = 11,307) of the study population without children. When the analysis was limited to women working at the WHI-OS baseline, the protective effect of working in period 1 was unchanged (Table 5, Sensitivity Analysis). The protective effect of working in period 4 was no longer statistically significant, but working during period 2, or the time of raising young children, was positively associated with physical limitations (RR = 1.07, CI: 1.02–1.13, Table 5).

## 4. Discussion

The results of our regression analysis demonstrated that over the course of their careers, women who held jobs with higher substantive complexity, requiring them to problem solve, make decisions, and think critically, had better physical functioning in later life than women whose jobs were not substantively complex. Although not mutually exclusive, women in jobs with high physical demands, which in the context of this analysis were related to the speed and coordination required to operate or maintain machinery, had a higher probability of physical limitations later in life. Social collaboration, though not significant after controlling for education, was also associated with a protective effect. The effect of substantive complexity and physical demand remained even after controlling for education and for income, suggesting that these effects are not explained by socioeconomic status.

Sensitivity analyses confirmed the ability of our results to hold true under various conditions. The fact that the estimates of association were relatively unchanged based on different cutoffs for categorizing exposure suggests that the results may be driven by women at the higher and lower ends of the range of work characteristics. For the outcome, however, point estimates changed based on the cutoff in physical functioning score, with stronger associations observed for the lower percentile cutoff in score. In other words, for more extreme outcomes or more severe limitations in physical functioning, there is a stronger association with work characteristics. This trend in the strength of associations supports the idea that there is a genuine relationship between work characteristics and physical functioning.

For women who self-identify as homemakers, the attenuation of the association between work characteristics and physical functioning suggests that perhaps for these women, the work atmosphere is a less important source of stress or relief because, despite holding jobs outside the home, they consider “homemaker” to be their primary job. The fact that working in the time period with older children in the house was positively associated with physical limitations only in this population suggests that work itself is a risk factor (regardless of work content) when women view work as secondary to being a homemaker. Among veterans, the most notable finding was the fact that many of the associations seen in the main analysis were not seen among veterans; this may be due to the lack of variability in exposure among this select group of women. Less than 3% of the study population self-identified as veterans, thus any associations were not statistically significant when the analysis was limited to this group. 

Finally, limiting the analysis to women actively working at baseline did not lead to a change in associations between work factors and physical functioning. This is important, since the presence of a healthy worker effect could suggest that women who leave the work force due to health issues might be more likely to leave physically demanding jobs or jobs that are less mentally satisfying and thus associations could be attributed to reverse causality. The fact that the associations between substantive complexity and physical demand of work with physical limitations were strengthened when limited to women working at the WHI-OS baseline implies that the association was not being driven by unhealthy women who had left the work force. 

The results for substantive complexity and physical demand are generally consistent with prior research. Previous studies have found that poor psychosocial working conditions, including low job control and monotonous work, increased frailty and the risk of poor health functioning and musculoskeletal problems, as well as other adverse health outcomes such as depression and hypertension [6,21,25,51,52,53,54]. Conversely, creative work has been associated with improved mental health and may even improve regulation of biological systems such as the hypothalamic-pituitary-adrenal (HPA) axis [55,56]. However, the substantive complexity of work has also been associated with health behaviors such as smoking [57] and alcohol intake [58] and may influence other health indicators such as hypertension [46]. The potentially complex pathway linking psychosocial work exposure and physical functioning may explain why the effect of substantively complex work was no longer statistically significant after accounting for potential mediators. Although our results suggest a somewhat more modest association between work characteristics and the risk of physical functioning limitations compared to previous studies with similar outcomes [6,25], the lower magnitude may be due to the fact that this was not an occupational cohort, and exposures to work characteristics had to be estimated across a wide variety of jobs and industries. Furthermore, physical functioning was assessed sometimes years after women last worked outside the home, and the effects of work characteristics may diminish over time. 

Physical demand or biomechanical strain at work has been associated with a variety of physical health problems [59,60]. A recent study concluded that occupational biomechanical strain is associated with physical functioning difficulties post-retirement in both men and women [61]. The association with such jobs may be the result of overuse or from accidents or injuries experienced on the job, but data are not available to assess the mechanism of disability. 

Social support at work has been found to have a positive effect on health, decreasing the risk of cardiovascular disease and depressive symptoms [9,62,63]. Furthermore, social support at work may buffer the effects of stress on emotional exhaustion and work-family conflict [20,64]. However, social collaboration as measured from these data represent the ability to work with others, maintaining composure, and being sensitive to their needs; this may not be equivalent to social support as measured in previous studies that is related to developing a rapport with coworkers during and outside of work [9,62,63].

Although the association between job characteristics and physical limitations was not modified by critical time period, we observed an overall beneficial effect of work during the time periods before and after children. Regardless of work characteristics, there appears to be a beneficial effect for women from early paid work before having children. Early work, which is observed in women who choose to delay childbearing and tend to have fewer children, has been associated with stronger attachment to the labor force and delayed retirement [65,66,67]. In our analysis, the relationship between early work and improved physical functioning persists even after controlling for education, income, and work characteristics, but not after controlling for later life mediators such as body mass index, smoking and alcohol use, suggesting that early work may influence life-long behaviors.

Both education and income were shown to be strong confounders of the effect of job characteristics on later life physical functioning and income may in fact be a partial mediator on the pathway from work characteristics to disability. This is not unexpected since we know that socioeconomic status (SES) is an important predictor of physical functioning [33,68,69] and occupation is intrinsically linked to education and income [70]. These components of SES are related to health through access to information and resources that promote health, including insurance, nutrition, housing, and recreation, as well as exposure to physical and psychosocial stressors [70]. There may also be other indicators that are important predictors of health but were unavailable in our data such as early-life SES (e.g., parental education) or neighborhood SES variables. Our inability to control for early-life indicators of SES may have important implications for our results. In particular, parental education has been shown to have an additive effect of women’s own education on physical functioning [68]. Given the strong positive association between beneficial job characteristics and additional education, this suggests that the observed associations may have been further attenuated if we were able to control for parental education. However, in prior research, occupation has been found to represent a distinct facet of SES [71] and job characteristics such as job strain and job control were associated with health outcomes independently of SES [72,73]. 

While use of exposure information from a job exposure matrix reduces differential misclassification or information bias related to self-report of job exposure [74], O*NET assumes a homogeneous distribution of job characteristics within job titles. Use of such a matrix contributes to measurement error because it fails to capture nuances in jobs that may be important when trying to describe the psychosocial work atmosphere and the internalization of such atmosphere by individuals. This may be particularly important for studies which attempt to use O*NET to identify work exposures in women. O*NET values were originally based on expert reviews but have been updated with surveys of random samples of employers and workers in selected jobs [40]. For jobs that are dominated by males, the levels of job characteristics may not be representative of those experienced by women in those fields. Women and men in the same job may not perform the same tasks and women are more likely to experience gender harassment or discrimination [75,76]. If job characteristics are based on interviews mainly among men, then the exposures among women may not be fully represented and there may be a higher degree of misclassification of exposures for women in male-dominated fields than in female-dominated fields. Furthermore, we were limited by the ability of the algorithm used to match job title with the appropriate SOC code; while the matching has been deemed sufficient at the 3-digit SOC level, there may be substantial misclassification at the 6-digit SOC level. In both instances, such measurement error was non-differential because misclassification occurred independently of the assessment of physical functioning and we expect that our results were biased towards the null and the true effects of job characteristics would be stronger than our results indicate. Even with these limitations in the data, factor analysis identified three components of work characteristics that could be estimated from O*NET data: substantive complexity, physical demands, and social collaboration. Substantive complexity is a concept of occupational conditions first identified by Kohn and more recently by Hadden et al. and Meyer et al., using O*NET data [41,77,78]. Although the variables which comprise substantive complexity here were slightly different due to our use of an updated O*NET database, the factors we identified are conceptually consistent with those previously identified [41,78], providing evidence of the construct validity of our exposure measures. 

An additional limitation is the fact that women only listed their three longest-held jobs instead of their full work history; there may be some bias in the jobs women chose to report. However, limiting the questionnaire to only three jobs simplified the task for participants and studies have shown that simple job information can be recalled with useful accuracy [79]. Finally, much of the information gathered in WHI, including the outcome physical functioning, was collected at baseline. Although women were asked to report jobs that they held prior to baseline and therefore prior to the assessment of physical functioning, the onset of physical limitations, disabilities, or other comorbidities and risk factors collected at the WHI-OS baseline cannot be assessed and may have occurred prior to starting work. In 2010, 30% of non-institutionalized adults 65 to 74 years old and 45% of adults age 75 or older had difficulties in physical functioning. The most common difficulties were standing for 2 h, walking a quarter of a mile, and stooping, bending, or kneeling [80]. Though prevalence of difficulties is not directly comparable with our outcomes, the types of difficulties reported are consistent with our results in which women with substantial limitations had self-reported limitations in vigorous activity, climbing several flights of stairs, bending, and walking 1 mile. We included hard exercise at age 18 to control in part for self-report of early life functioning.

## 5. Conclusions

Our study is one of the first to explore the effects of psychosocial work exposure throughout the life course on later life physical functioning in a large population of US women across different sectors of the work force, and to explore the effects of work during critical time periods in adulthood. Although further studies are needed that longitudinally follow women throughout their careers and collect concurrent health and risk factor information, there is consistent information in the literature that is supported by our results to show the modest beneficial effects of substantively complex work. Given the large study population and breadth of occupations, we believe our findings are generalizable to US women as a whole, and employers should enable workers more opportunities to express ideas, solve problems, and learn new skills; such opportunities may improve the psychosocial work atmosphere. Additionally, physically demanding jobs may be especially detrimental to women in male-dominated, manual occupations when machines and protective equipment are not ergonomically designed for women [81,82]; employers should make efforts to ensure that equipment is appropriate for all workers. Improvement in working conditions across all sectors is imperative at a time when the workforce is aging rapidly. Efforts to improve the health of workers can not only help keep Americans working longer, but can also help to reduce the healthcare costs and burden of physical disability in the aging population. 

## Figures and Tables

**Table 1:** Factor analysis of Occupational Information Network (O*NET) variables to identify categories of job characteristics (O*NET version 18.1).

Factor	Attribute	Variable Loading Value
Substantive Complexity		
	Complex problem solving	0.95
	Active learning	0.94
	Judgment and decision making	0.93
	Deductive reasoning	0.93
	Analyzing data or information	0.93
	Critical thinking	0.93
	Inductive reasoning	0.92
	Interpreting the meaning of information for others	0.92
	Systems analysis	0.91
	Systems evaluation	0.90
	Making decisions and solving problems	0.88
	Monitoring	0.88
	Learning strategies	0.87
	Reading comprehension	0.86
	Information ordering	0.86
	Writing	0.86
	Category flexibility	0.85
	Updating and using relevant knowledge	0.84
	Instructing	0.84
	Written comprehension	0.84
	Memorization	0.83
	Developing objectives and strategies	0.83
	Fluency of ideas	0.83
	Processing information	0.83
	Written expression	0.83
	Getting information	0.81
	Identifying objects, actions, and events	0.80
	Originality	0.80
Physical Demand		
	Rate control	0.99
	Response orientation	0.99
	Reaction time	0.98
	Controlling machine and processes	0.97
	Operation and control	0.97
	Repairing and maintaining mechanical equipment	0.96
	Equipment maintenance	0.91
	Multilimb coordination	0.90
	Repairing	0.88
	Operating vehicles, mechanized devices, or equipment	0.88
	Wrist-finger speed	0.87
	Control precision	0.87
	Sound localization	0.85
	Exposed to hazardous equipment	0.82
	Performing general physical activities	0.80
	Handling and moving objects	0.80
Social Collaboration		
	Self control	0.96
	Concern for others	0.94
	Social orientation	0.92
	Cooperation	0.82
	Co-workers, social service, and moral values	0.80

**Table 2:** Participant characteristics, Women’s Health Initiative Observational Study, 1993–1998 (*n* = 79,147).

	Overall	Factor 1: Substantive Complexity (≥0.48)	Factor 2: Physical Demand (≥0.18)	Factor 3: Social Collaboration (≥0.75)
Characteristic (*n* (col %))				
Age (mean ± SD)	63.4 ± 7.3	62.9 ± 7.3	63.5 ± 7.4	63.3 ± 7.3
Race/Ethnicity (*n* = 78,955)		*	*	*
American Indian or Alaskan Native	284 (0.4)	108 (0.3)	177 (0.5)	128 (0.3)
Asian or Pacific Islander	2208 (2.8)	1165 (3.0)	1098 (2.8)	1092 (2.8)
Black	5611 (7.1)	2753 (7.0)	3094 (7.8)	3092 (7.9)
Hispanic/Latino	2310 (2.9)	902 (2.3)	1330 (3.4)	1100 (2.8)
White	67,711 (85.8)	34,255 (86.6)	33,402 (84.5)	33,585 (85.4)
Other	831 (1.1)	369 (0.9)	445 (1.1)	354 (0.9)
Marital Status (*n* = 78,828)		*	*	*
Never married	4032 (5.1)	2636 (6.7)	1832 (4.6)	2288 (5.8)
Divorced or separated	12,394 (15.7)	6440 (16.3)	6222 (15.8)	5809 (14.8)
Widowed	13,098 (16.6)	5810 (14.7)	6996 (17.7)	6130 (15.6)
Married at baseline	47,978 (60.9)	23,895 (60.5)	23,730 (60.1)	24,448 (62.2)
Marriage-like relationship	726 (1.7)	726 (1.8)	686 (1.7)	614 (1.6)
Birth Region (*n* = 78,649)		*	*	*
Northeast	22,266 (28.3)	11,517 (29.2)	10,774 (27.4)	11,355 (29.0)
Midwest	23,787 (30.2)	11,996 (30.4)	12,060 (30.7)	12,037 (30.7)
South	16,669 (21.2)	7965 (20.2)	8312 (21.1)	8184 (20.9)
West	10,796 (13.7)	5377 (13.6)	5399 (13.7)	5179 (13.2)
Not born in US	5131 (6.5)	2579 (6.5)	2801 (7.1)	2415 (6.2)
Education (*n* = 78,573)		*	*	*
Less than high school	3127 (4.0)	544 (1.4)	2457 (6.2)	969 (2.5)
High school graduate	40,731 (51.8)	13,572 (34.5)	22,651 (57.6)	15,796 (40.3)
College degree or higher	34,715 (44.2)	25,244 (64.1)	14,241 (36.2)	22,410 (57.2)
Body Mass Index Category (*n* = 78,241)		*	*	*
Underweight (<18.5)	932 (1.2)	521 (1.3)	431 (1.1)	473 (1.2)
Normal (18.5–24.9)	31,593 (40.4)	16,901 (43.1)	14,960 (38.2)	16,258 (41.7)
Overweight (25.0–29.9)	26,484 (33.9)	13,014 (33.2)	13,251 (33.8)	13,218 (33.9)
Obese (30.0+)	19,232 (24.6)	8744 (22.3)	10,546 (26.9)	9024 (23.2)
Income (*n* = 75,994)		*	*	*
Less than $20 K	10,833 (14.3)	3400 (8.9)	6896 (18.1)	4389 (11.6)
$20,000–34,999	16,969 (22.3)	7108 (18.6)	9004 (23.6)	7807 (20.6)
$35,000–49,999	15,056 (19.8)	7678 (20.1)	7388 (19.4)	7715 (20.4)
$50,000–74,999	15,363 (20.2)	8982 (23.6)	6886 (18.1)	8397 (22.2)
$75 K+	15,688 (20.6)	10,100 (26.5)	6766 (17.8)	8530 (22.5)
Don’t know	2085 (2.7)	870 (2.3)	1158 (3.0)	1023 (2.7)
Number of live births		*	*	*
0	11,307 (14.3)	6787 (17.2)	5130 (12.9)	5994 (15.2)
1	7175 (9.1)	3701 (9.4)	3555 (8.9 )	3416 (8.9)
2–4	51,263 (64.8)	25,038 (64.0)	25,688 (64.6)	25,504 (64.8)
5+	9403 (11.9)	3754 (9.5)	5395 (13.6)	4453 (11.3)
Smoking status (*n* = 78,329)		*	*	*
Never smoked	39,389 (50.3)	19,782 (50.4)	19,611 (50.0)	20,194 (51.7)
Past smoker	34,084 (43.5)	17,320 (44.1)	16,965 (43.3)	16,650 (42.7)
Current smoker	4856 (6.2)	2143 (5.5)	2634 (6.7)	2182 (5.6)
Alcohol intake (*n* = 78,802)		*	*	*
Non-drinker	8147 (10.3)	3564 (9.0)	4399 (11.2)	4122 (10.5)
Past drinker	14,421 (18.3)	6321 (16.0)	7900 (20.0)	6795 (17.3)
<1 drink/month	9272 (11.8)	4391 (11.1)	4871 (12.4)	4534 (11.6)
<1 drink/week	15,929 (20.2)	8234 (20.9)	7829 (19.9)	8151 (20.8)
1 to <7 drinks/week	20,703 (26.3)	11,287 (28.6)	9680 (24.5)	10,557 (26.9)
7+ drinks/week	10,330 (13.1)	5683 (14.4)	4770 (12.1)	5102 (13.0)
Hard exercise at age 18 (*n* = 76,844)	34,277 (44.6)	17,329 (44.8)	17,442 (45.5) *	17,279 (45.0) *
Hard exercise at age 35 (*n* = 77,106)	34,341 (44.5)	16,968 (43.7) *	17,539 (45.6) *	17,055 (44.3)
Hard exercise at age 50 (*n* = 77,654)	30,718 (39.6)	15,694 (40.2) *	15,360 (39.6)	15,574 (40.2) *
Depressive symptoms (shortened CES-D ^†^ ≥ 0.06) (*n* = 77,647)	8446 (10.9)	3691 (9.5) *	4545 (11.7) *	3838 (9.9) *
Presence of Social support (*n* = 77,521)	41,919 (54.1)	21,514 (55.3) *	20,460 (52.8) *	21,698 (56.1) *
Presence of Social strain (*n* = 77,941)	44,522 (57.1)	21,778 (55.7) *	22,679 (58.2) *	21,815 (56.1) *
Presence of comorbidities ^a^	63,686 (80.5)	31,585 (79.7) *	32,181 (81.2) *	31,619 (80.2) *
Physical limitations ^§^	18,718 (23.7)	7998 (20.2) *	10,330 (26.1) *	8708 (22.1) *

* Distributions differ across levels of factors (*p* < 0.05); ^†^ CES-D: Center for Epidemiologic Studies Depression Scale; ^a^ Cardiovascular disease, hypertension, arthritis, osteoporosis, Alzheimer’s, cancer, emphysema, hip fracture after age 55, myocardial infarction, stroke, diabetes, falls in the last year; ^§^ PF Score ≤75.

**Table 3:** Job characteristics, Women’s Health Initiative Observational Study, 1993–1998 (*n* = 79,147).

	Overall	Factor 1: Substantive Complexity (≥0.48)	Factor 2: Physical Demand (≥0.18)	Factor 3: Social Collaboration (≥0.75)
Characteristic (*n* (col %))				
Years worked (mean ± SD)	22.7 ± 10.9	23.9 ± 10.4	22.5 ± 11.1	23.5 ± 10.9
Number of jobs listed		*	*	*
1	11,926 (15.1)	6352 (16.0)	5253 (13.3)	6767 (17.2)
2	15,910 (20.1)	7734 (19.5)	7929 (20.0)	8029 (20.4)
3	25,680 (64.8)	25,562 (64.5)	26,457 (66.7)	24,641 (62.5)
Critical periods of work (not mutually exclusive, shown as *n* (%))				
Before children	38,894 (49.1)	20,613 (53.0) *	18,376 (46.4) *	20,730 (52.6) *
With young children	45,916 (58.0)	22,778 (57.5) *	23,288 (58.8) *	23,777 (60.3) *
With older children	58,123 (73.4)	29,056 (73.3)	29,442 (74.3) *	28,948 (73.4)
After children are adults	54,333 (68.7)	26,681 (67.3) *	27,702 (69.9) *	26,354 (66.8) *
Work status		*	*	*
Actively working at baseline	33,779 (42.7)	17,675 (44.6)	17,126 (43.2)	16,520 (41.9)
Recently retired (<10 years)	26,528 (33.5)	13,551 (34.2)	13,063 (33.0)	13,367 (33.9)
Retired 10+ years	18,840 (23.8)	8422 (21.2)	9450 (23.8)	9550 (24.2)
Job type ^†^		*	*	*
Male-dominated, manual	7990 (10.1)	1142 (2.9)	7463 (18.8)	1170 (3.0)
Female-dominated, high SES ^§^	8673 (11.0)	4561 (11.5)	7863 (19.8)	7429 (18.8)
Female-dominated, low SES	1917 (2.4)	109 (0.3)	1692 (4.3)	381 (1.0)
Other	60,567 (76.5)	33,836 (85.3)	22,621 (57.1)	30,457 (77.2)
Homemakers (*n* = 75,861)	19,345 (25.5)	7602 (19.9) *	10,627 (28.0) *	9,251 (24.4) *
Veterans (*n* = 74,524)	1978 (2.7)	1102 (2.9) *	1115 (2.9) *	1093 (3.0) *

* Distributions differ across levels of factors (*p* < 0.05); ^†^ Male-dominated, manual labor jobs includes protective service, building, and grounds cleaning and maintenance, farming and fishing, construction and extraction, installation and maintenance, production, and transportation and material moving occupations. Female-dominated, higher SES jobs include healthcare practitioners and healthcare support occupations. Female-dominated, low SES jobs include food preparation and service occupations. Other jobs consist of all other occupational categories, including clerical jobs and teachers. ^§^ SES: Socioeconomic Status.

**Table 4:** Association between physical limitations (physical functioning score <75) and job characteristic, Women’s Health Initiative Observational Study, 1993–1998 (*n* = 79,147).

		Risk Ratio (95% Confidence Interval)		
Model	Model Fit (QICu)	Substantive Complexity	Physical Demand	Social Collaboration
1: Crude ^1^	118913	0.78 (0.76–0.80) *	1.14 (1.11–1.17) *	0.95 (0.93–0.98) *
2: Crude + confounders ^2^	110170	0.81 (0.79–0.84) *	1.12 (1.10–1.15) *	0.94 (0.92–0.97) *
3: Model 2 + education	109219	0.94 (0.91–0.96) *	1.09 (1.06–1.12) *	1.01 (0.98–1.03)
4: Model 3 + income	104786	0.96 (0.93–0.98) *	1.08 (1.05–1.11) *	1.00 (0.98–1.03)
5: Model 4 + mediators ^3^	95959	0.97 (0.95–1.00)	1.04 (1.01–1.06) *	1.00 (0.98–1.03)
Working at baseline ^4^	37004	0.93 (0.88–0.98) *	1.10 (1.05–1.15) *	1.01 (0.96–1.06)
Homemakers ^5^	29907	0.96 (0.91–1.01)	1.07 (1.01–1.12) *	0.98 (0.93–1.03)

* Statistically significant at α = 0.05. ^1^ Controlling for work during critical time periods; ^2^ Age, birth region, race/ethnicity, marital status, and exercise at age 18; ^3^ Body mass index category, retirement status, presence of comorbidities, corticosteroid use, social support, social strain, presence of depressive symptoms, energy expenditure per week, smoking status, alcohol intake, number of children; ^4^ Model 3 limited to actively working at baseline (*n* = 32,242); ^5^ Model 3 limited to self-identified homemakers (*n* = 18,526).

**Table 5:** Association between physical limitations (physical functioning score <75) and crucial period, Women’s Health Initiative Observational Study, 1993–1998 (*n* = 79,147).

	Risk Ratio (95% Confidence Interval)			
	Critical Periods			
Model	Before Children	With Young Children	With Older Children	After Children
1: Crude ^1^	0.89 (0.86–0.91) *	1.00 (0.97–1.03)	0.90 (0.87–0.94) *	0.92 (0.89–0.95) *
2: Crude + confounders ^2^	0.88 (0.86–0.91) *	1.00 (0.97–1.03)	1.02 (0.98–1.06)	0.86 (0.83–0.89) *
3: Model 2 + education	0.92 (0.89–0.94) *	1.01 (0.98–1.04)	1.03 (0.99–1.07)	0.87 (0.84–0.90) *
4: Model 3 + income	0.93 (0.90–0.95) *	1.01 (0.99–1.04)	1.01 (0.98–1.05)	0.87 (0.84–0.90) *
5: Model 4 + mediators ^3^	1.00 (0.97–1.03)	0.98 (0.95–1.00)	1.01 (0.97–1.05)	0.99 (0.95–1.03)
Working at baseline ^4^	0.91 (0.86–0.96) *	1.07 (1.02–1.13) *	0.96 (0.89–1.03)	0.93 (0.86–1.01)

* Statistically significant at α = 0.05. ^1^ Controlling for work during critical time periods; ^2^ Age, birth region, race/ethnicity, marital status, and exercise at age 18; ^3^ Body mass index category, retirement status, presence of comorbidities, corticosteroid use, social support, social strain, presence of depressive symptoms, energy expenditure per week, smoking status, alcohol intake, number of children; ^4^ Model 3 limited to actively working at baseline (*n* = 32,242).
